# Predictors of nurse and midwife performance in a Ugandan district: a mixed-methods study of individual, organizational, and community factors

**DOI:** 10.1186/s12913-026-14866-8

**Published:** 2026-06-08

**Authors:** Edmonton Acheka, John Paul Byagamy, Richard Kajura, Elizeus Rutebemberwa

**Affiliations:** 1 Department of Health Services, Lira District Local Government, Lira, Uganda; 2 School of Public Health, College of Health Sciences, Makerere University, Kampala, Uganda; 3 Department of Environmental Health & Disease Control, Faculty of Public Health, Lira University, Lira City, Uganda

**Keywords:** Health workforce performance, Nurses, Midwives, Professional accountability, Health systems strengthening, Uganda, Mixed-methods research

## Abstract

**Background:**

A well-performing health workforce, defined as one that is available, competent, productive, and responsive to patient needs, is central to achieving Sustainable Development Goals SDG 3.8 on Universal Health Coverage. In Uganda, district-level health performance reports have indicated persistent challenges. Addressing health workforce performance gaps is therefore a critical health systems issue for achieving district-level health targets. This study assessed factors affecting the performance of professional nurses and midwives defined as enrolled, registered, and degree-level practitioners licensed by the Uganda Nurses and Midwives Council, in Lira District, Northern Uganda.

**Methods:**

A cross-sectional convergent parallel mixed-methods study was conducted from April 2017 to May 2018. A structured questionnaire was administered to 156 randomly selected nurses (*n* = 98) and midwives (*n* = 58) across all government (*n* = 24) and private-not-for-profit (*n* = 6) facilities. Performance was measured across four dimensions: competency, productivity, availability, and responsiveness. Principal Component Analysis (PCA) reduced independent variables. Linear regression identified predictors of performance. Qualitative data from 20 key informant interviews (KIIs) and three Focus Group Discussions (*n* = 30) were analyzed thematically.

**Results:**

The majority of respondents were female (83.3%), certificate holders (72.4%), and had 1–10 years of experience (54.5%). PCA yielded six components (C1-C6) explaining 85.9% of variance. Performance levels varied across dimensions: half of the respondents (50.0%) had competency scores in the 0–50% range, while productivity (60.3%), availability (51.3%), and responsiveness (75.0%) scores were predominantly in the 51–75% range. Linear regression identified distinct predictors for each dimension: Competency was significantly predicted by C1 (Poor Clinical Practice, β=-0.010, *p* = 0.012), C2 (Adherence to Systems, β = 0.066, *p* < 0.001), C3 (Organizational Commitment, β = 0.039, *p* < 0.001), and C4 (Participatory Environment, β = 0.080, *p* < 0.001). Productivity was predicted by C1 (β=-0.029, *p* < 0.001), C2 (β = 0.030, *p* = 0.006), C3 (β = 0.044, *p* = 0.005), and C4 (β = 0.131, *p* < 0.001). Availability was predicted by C2 (β = 0.090, *p* < 0.001), C4 (β = 0.131, *p* < 0.001), C5 (Unmet Training Needs, β=-0.060, *p* < 0.001), and C6 (Politicized Recruitment, β=-0.020, *p* = 0.004). Responsiveness was predicted by C2 (β = 0.070, *p* < 0.001), C4 (β = 0.139, *p* < 0.001), and C5 (β=-0.047, *p* < 0.001). Qualitative findings from 20 key informant interviews and 3 FGDs revealed key contextual factors: poor health-seeking behaviors (mentioned in 85% of FGDs), political interference in recruitment/promotion (reported by 70% of key informants), lack of community ownership of health facilities (discussed in 80% of FGDs), and negative staff attitudes (raised in 85% of FGDs).

**Conclusions:**

Individual factors (clinical practices, motivation), organizational factors (working environment, systems adherence, resource availability), and political economy factors (political interference in recruitment/promotion) significantly predict nurse and midwife performance across all dimensions. Community-level factors (poor health-seeking behaviors, negative mutual perceptions) further undermine performance and service utilization. A multi-level intervention addressing individual capacity, organizational support, depoliticized human resource management, and community-health system linkages is urgently needed to improve workforce performance and service delivery. This requires coordinated action from national policymakers, district managers, facility leaders, and communities.

**Supplementary Information:**

The online version contains supplementary material available at 10.1186/s12913-026-14866-8.

## Background

The performance of the health workforce is a critical determinant of health system effectiveness and a cornerstone for achieving the Sustainable Development Goals (SDGs), particularly SDG 3.8 on achieving Universal Health Coverage (UHC), which aims to ensure access to quality essential healthcare services without financial hardship [1, 2]. Nurses and midwives constitute over 60% of the health workforce in many countries, including Uganda, playing a pivotal role in primary healthcare delivery, especially in underserved areas [3, 4]. However, Uganda’s nurse and midwife density of 1.5 per 1,000 population falls substantially below the WHO threshold of 4.45 per 1,000 needed to achieve UHC, and compares unfavorably with high-income countries such as the United Kingdom (8.2 per 1,000) and the United States (14.5 per 1,000) [5]. Globally, there is a recognized crisis in the health workforce, with a projected shortfall of 18 million health workers by 2030, predominantly in low- and middle-income countries [1, 5].

**Fig. 1:**
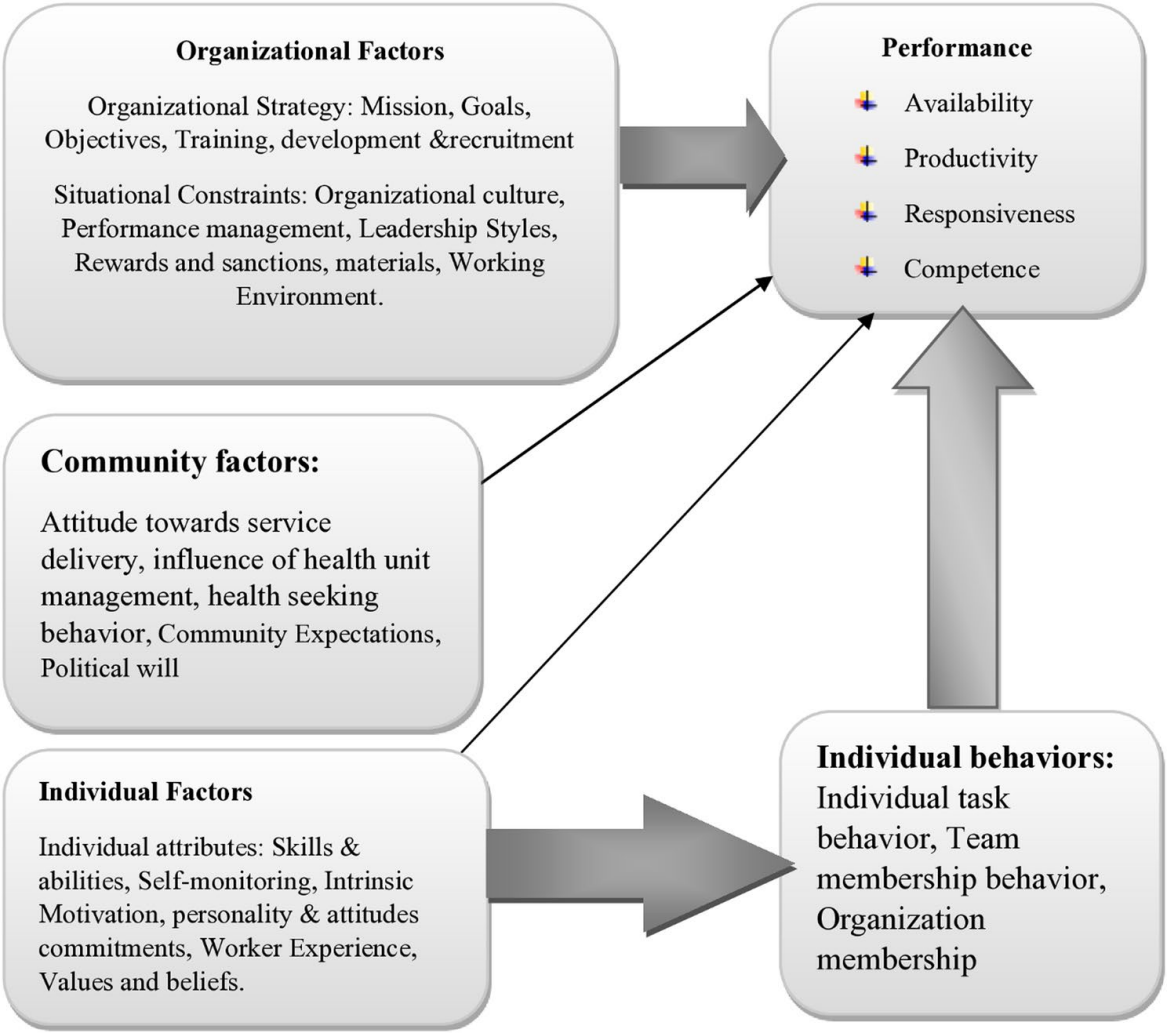
Conceptual Framework of factors affecting the performance of professional nurses and midwives in Lira District, Uganda. (Adapted from Noe et al., 2008 [25])

Performance, conceptualized as a combination of availability, competency, productivity, and responsiveness, is influenced by a complex interplay of individual, organizational, and community-level factors (Fig. 1) [6, 7]. Individual factors such as skills, motivation, job satisfaction, and remuneration are well-documented determinants [8, 9]. Organizational factors, including leadership, supervision, resource availability (drugs, equipment), and the working environment, create the context within which care is delivered [10, 11]. Furthermore, community perceptions, health-seeking behaviors, and the relationship between communities and health workers significantly impact service utilization and provider morale [12, 13].

In Uganda, decentralization was implemented in the 1990s to improve service delivery by transferring authority to district governments. However, concerns have been raised about its impact on health workforce management, including: fragmentation of human resource functions across 135 districts, inconsistent application of staffing norms, delayed salary payments, politicization of recruitment decisions, and weakened supervision due to competing priorities at the district level [14, 15]. Lira District in Northern Uganda has consistently shown poor performance in national health league tables, with deteriorating indicators in immunization, antenatal care, and maternal health outcomes between 2014/15 and 2016/17 [16]. In Uganda, the Ministry of Health publishes annual Health Sector Performance Reports that rank districts using a league table approach based on key indicators, including immunization coverage (target: >90%), antenatal care attendance (target: 4 visits at > 95%), and institutional delivery rates (target: >80%). Lira District consistently ranked in the bottom quartile of Ugandan districts between 2014/15 and 2016/17, with immunization coverage declining from 72% to 58% against the national target of > 90%, and institutional delivery rates stagnating at 45% against the national target of > 80% [16]. Concurrent district health information systems (DHIS) reports highlight high absenteeism (estimated at 30–40%), low morale, and poor motivation among health workers in the district [17].

Despite these challenges, there is limited localized evidence on the specific factors affecting the performance of nurses and midwives, who form the backbone of service delivery. Existing studies in Uganda have often focused on broader health system issues such as health financing mechanisms [18], decentralization policy implementation [14], or specific cadres like doctors [19], leaving a gap in understanding the drivers of performance for this critical group at the district level [18, 19]. Studies that have examined nursing performance have typically focused on clinical competency alone rather than the multi-dimensional nature of performance (availability, productivity, responsiveness) [20], and few have explored how community-level factors interact with individual and organizational determinants in shaping overall performance, as recent studies continue to demonstrate [21, 22]. This study, therefore, sought to determine the individual, organizational, and community factors affecting the performance of professional nurses and midwives in health facilities in Lira District, Uganda. The findings aim to inform targeted interventions and policies to strengthen the nursing and midwifery workforce, ultimately contributing to improved health outcomes.

## Methods

### Study design and setting

**Fig. 2:**
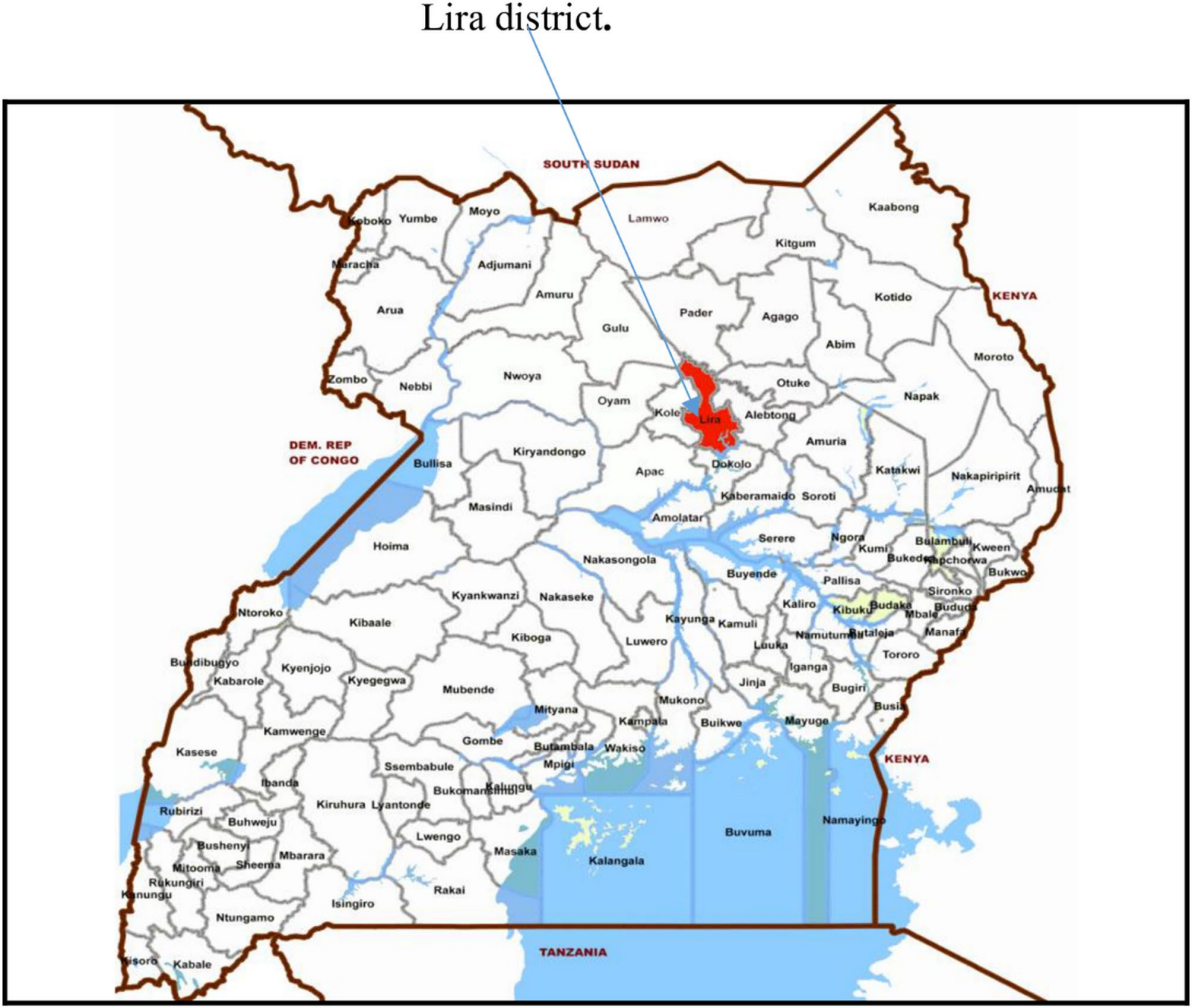
Map of Uganda showing Lira district. (adapted from District Biostatistician Lira)

A convergent parallel mixed-methods design was conducted from April 2017 to May 2018 in Lira District, Northern Uganda (Fig. 2). This design was chosen because it allows for simultaneous collection of quantitative and qualitative data, enabling triangulation of findings and providing both breadth (through the survey) and depth (through interviews and FGDs) in understanding the complex, multi-level factors affecting health worker performance [23]. The quantitative component assessed the prevalence and strength of associations between individual, organizational factors and performance, while the qualitative component explored the contextual mechanisms and lived experiences underlying these associations (Supplementary file 1).

**Fig. 3:**
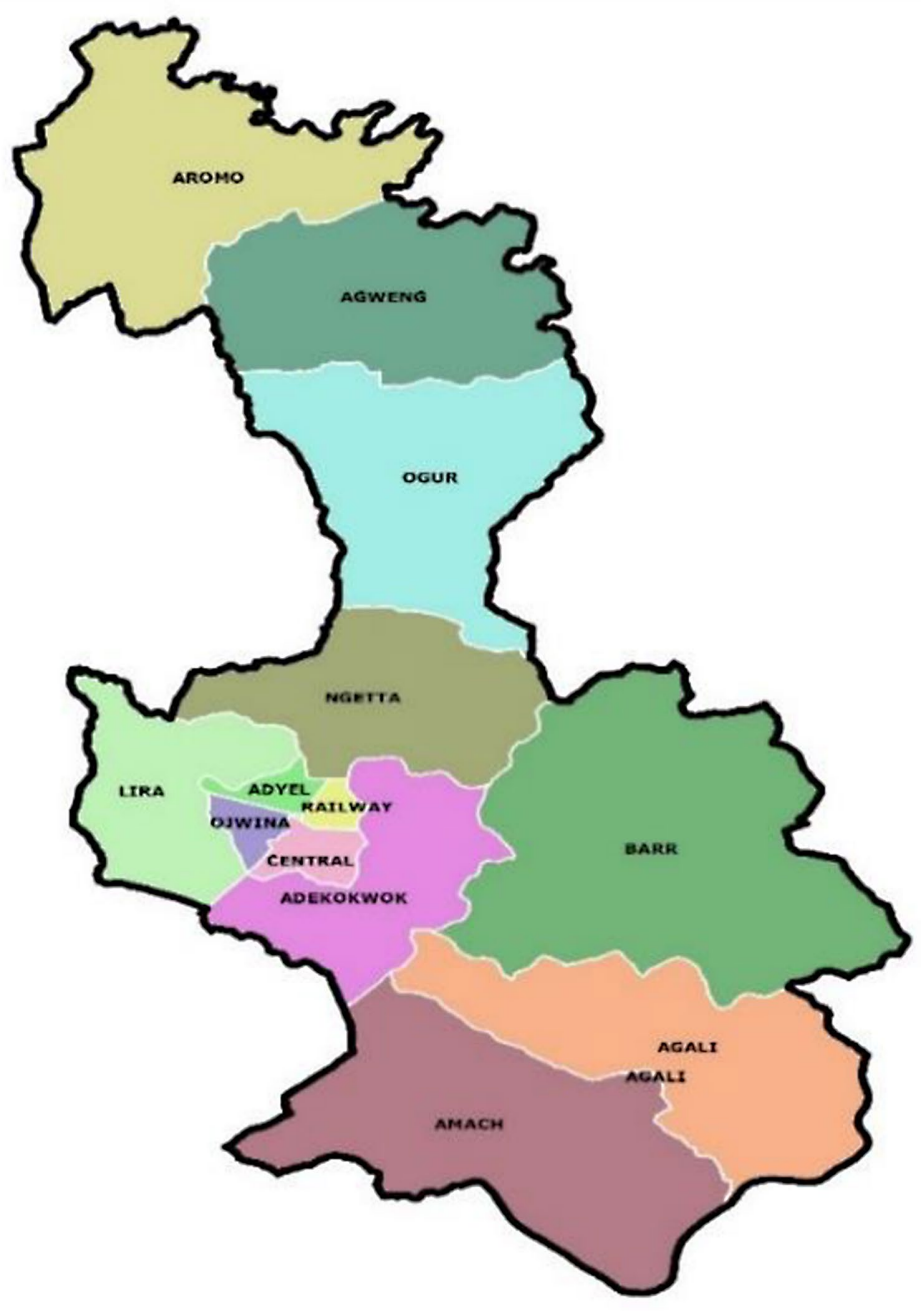
Detailed map of the Lira district. (adapted from District Biostatistician Lira)

Lira District is located in the Lango sub-region (Fig. 3) and has one regional referral hospital, 3 Health Centre IVs, 17 Health Centre IIIs, and 10 Health Centre IIs, serving a population of approximately 445,975 [based on 2014 census projections]. In Uganda’s health system structure, Health Centre IIs provide outpatient care and community outreach (serving populations of 5,000–10,000); Health Centre IIIs provide inpatient care, maternity services, and basic laboratory services (serving 20,000–30,000); Health Centre IVs provide emergency surgery, blood transfusion, and supervision of lower-level facilities (serving 100,000+); and Hospitals provide comprehensive specialist services [24]. According to Uganda’s staffing norms, Health Centre IIs are primarily intended to be staffed by enrolled nurses (certificate level) rather than professional nurses/midwives. However, during our facility mapping, we found that 8 of the 10 Health Centre IIs had at least one professional nurse or midwife posted there, often due to: (1) shortage of enrolled nurses leading to deployment of available professional staff, (2) political pressure to place professional staff in community facilities, and (3) health workers choosing to work in lower-level facilities closer to their home communities. These facilities were therefore included in the sampling frame. A detailed description of facility classifications and service packages is provided in Supplementary file 2.

### Conceptual framework

This study was guided by an adapted strategic performance model [25], which posits that individual attributes and behaviors, shaped by organizational strategy and constraints, determine performance outcomes, with community factors acting as an external influence. The framework (Fig. 1) illustrates the relationships between the independent variables (Individual Factors, Organizational Factors, Community Factors) and the dependent variable (Performance of nurses and midwives, measured by Availability, Competency, Productivity, and Responsiveness). This model is appropriate for health services research as it explicitly links individual-level performance to organizational strategy and external contextual factors, providing a systems-level understanding of workforce performance.

### Study population and sampling

The study population comprised all professional nurses and midwives (enrolled, registered, and degree-level) working in Lira District’s public and private-not-for-profit (PNFP) health facilities. All 354 professional nurses and midwives working in the district during the study period were eligible to participate.

#### Quantitative sample

The sample size was calculated using the Kish Leslie formula for a finite population [26]. With an estimated 354 professional nurses/midwives (76% of the health workforce), a 95% confidence level, a 5% margin of error, and a design effect of 1, a minimum sample of 156 was required. A comprehensive list of all 354 eligible nurses and midwives was obtained from the District Health Office human resources database. This list was verified through: (1) cross-checking with facility in-charge reports from each health facility, (2) telephone confirmation with a random sample of 50 staff (10% of the list) to confirm their current employment status and location, and (3) during data collection visits, research assistants confirmed the presence of selected participants and updated the list for any discrepancies (e.g., staff on transfer, leave, or who had left employment).

Participants were selected using simple random sampling through the lottery method. Each person was assigned a unique number from 001 to 354. These numbers were written on identical slips of paper, folded uniformly, and placed in a container. A research assistant not involved in the study drew 156 slips from the container without replacement. The individuals corresponding to the drawn numbers were then invited to participate in the study. In cases where a selected individual was unavailable (e.g., on leave, transferred), an alternative was randomly drawn using the same procedure.

#### Qualitative sample

Purposive sampling was used to select 20 key informants interviews (KIIs), comprising: 5 District Health Office staff (District Health Officer, Assistant DHO-Nursing, Biostatistician, Health Educator, Human Resource Officer); 8 health facility in-charges (4 from government facilities, 2 from PNFP facilities, 2 from the regional referral hospital); 4 supervisors (2 senior nursing officers, 2 hospital matrons); and 3 district-level health managers (from the Health Sub-District coordination offices). It is important to note that the 20 key informants were purposively selected from supervisory and management positions and were not part of the 156 survey respondents, who were frontline nurses and midwives. This ensured no overlap between quantitative and qualitative samples and allowed each method to capture distinct perspectives.

Three Focus Group Discussions (FGDs), each with 10 participants, were held across the three Health Sub-Districts (Erute North, Erute South, and Lira Municipal). FGD participants included Health Unit Management Committee members, local council leaders, and community opinion leaders, current service providers (nurses and midwives) were excluded from FGDs to minimize bias and encourage community members to speak freely about their experiences with health services. However, service provider perspectives were extensively captured through the 12 key informant interviews conducted with facility in-charges, supervisors, and senior nursing staff, as well as through the quantitative survey (*n* = 156).

This approach ensured that both provider and community perspectives were gathered through appropriate methods: individual in-depth interviews for providers (allowing confidential reflection) and community FGDs (facilitating group interaction and shared experiences). FGD participants included: Health Unit Management Committee members (*n* = 12, 4 per FGD), local council leaders (*n* = 9, 3 per FGD), and community opinion leaders, including religious leaders, teachers, and traditional birth attendants (*n* = 9, 3 per FGD). A detailed breakdown of qualitative participants is provided in Supplementary Table S2.

### Data collection

**Quantitative data** were collected using a structured, pre-tested questionnaire adapted from a validated tool used in a prior Ugandan study on health worker performance [20]. The original tool was developed by Lutwama (2011) for assessing health worker performance in decentralized Ugandan health services and had been validated through expert review, pre-testing, and reliability testing (Cronbach’s alpha > 0.8 for all sub-scales). For this study, we adapted the tool to the Lira District context by: (1) conducting cognitive interviews with 5 nurses to ensure question comprehension; (2) adding context-specific items based on preliminary key informant discussions; (3) pre-testing the revised tool with 20 nurses in a neighboring district (Apac) to assess clarity, length, and cultural appropriateness. The final tool is provided in full in supplementary file 1.

Reliability was reassessed in our sample, with all scales showing good to excellent internal consistency: Individual Factors (α = 0.971), Organizational Factors (α = 0.943), and Performance Dimensions (α = 0.853). The questionnaire had four sections: socio-demographics, individual factors (attributes and behaviors), organizational factors (strategy and constraints), and performance dimensions (dependent variable). Performance was operationalized through 46 items across four sub-scales: Competency (9 items), Productivity (10 items), Availability (17 items), and Responsiveness (10 items). Responses were on a 5-point Likert scale (1 = Strongly Disagree to 5 = Strongly Agree). These items measured self-reported performance in routine service delivery tasks relevant to frontline nursing and midwifery roles (Supplementary file 1). Quantitative data were collected by EA and two trained research assistants (both with Bachelor’s degrees in Nursing and experience in health facility surveys). Research assistants received two days of training on the study protocol, ethical conduct, and questionnaire administration. EA supervised all data collection and conducted spot-checks on 10% of completed questionnaires for quality assurance.

**Qualitative data** were collected using interview guides for key informants and FGDs by EA (who conducted all KIIs) and co-facilitated by a research assistant for FGDs (one facilitated while the other took notes and managed audio recording). Themes explored included perceptions of performance, influencing factors, and community-health worker interactions (Supplementary file 1). All sessions were audio-recorded, transcribed verbatim, and supplemented with field notes. Transcription was performed verbatim in Luo (the local language) by two trained research assistants with backgrounds in social sciences and experience in qualitative research. Transcripts were then translated into English by a third research assistant fluent in both languages.

To ensure accuracy and transparency in quote reporting: quotes were transcribed verbatim; any modifications for clarity are indicated with brackets [ ], and original recordings are available for verification. Field notes documenting observations of facility environments, non-verbal cues, and contextual factors were integrated with transcripts during analysis. Field notes were written by EA immediately following each KII and FGD session and were typed and stored as separate documents linked to each transcript.

### Measurement of variables

#### Dependent variable

Performance was a composite average score derived from the four dimensions. Sub-scale scores were also analyzed independently. PCA was not applied to performance items because performance was conceptualized a priori based on the four theoretically-derived dimensions (competency, productivity, availability, responsiveness) from the adapted Noe et al. [25] framework. Maintaining these distinct dimensions allowed us to examine how the PCA-derived components predicted each aspect of performance separately, providing more nuanced insights than a single composite score would allow.

#### Independent variables

These included socio-demographic variables (age, sex, education, experience, facility level, location) and composite scores for *Individual Factors* (22 items) and *Organizational Factors* (23 items) derived from the Likert-scale items.

### Data quality control

The questionnaire was pre-tested, and reliability was assessed using Cronbach’s Alpha. All scales showed good to excellent internal consistency: Individual Factors (α = 0.971), Organizational Factors (α = 0.943), and Performance Dimensions (α = 0.853). Face and content validity were ensured through expert review by supervisors.

### Data analysis

**Quantitative data** were analyzed using STATA 14. Descriptive statistics summarized background characteristics. Principal Component Analysis (PCA) with varimax rotation was used for data reduction on the 45 items from individual and organizational factors. Components with eigenvalues > 1 were retained (Kaiser’s criterion) [27]. For interpreting the rotated component matrix, we used a cutoff of |0.28| for factor loadings, which corresponds to a sample size of 150, providing statistical significance at *p* < 0.05 with 80% power [28]. Loadings below this threshold are considered negligible and are not reported in the summary table (Table 3), though the full matrix with all loadings is available in Supplementary file 3. Pearson correlation assessed relationships between components and performance dimensions. Multiple linear regression models identified predictors of each performance dimension and overall performance, with statistical significance set at *p* < 0.05.

**Qualitative data** were analyzed using thematic content analysis following the framework approach [29]. Two researchers (EA and JPB) independently coded the first three transcripts to develop an initial coding framework. Codes were compared, discussed, and refined through consensus. The remaining transcripts were coded by EA using NVivo 11 software, with regular peer debriefing sessions with JPB and RK to review coding decisions and emerging themes. Both coders had training in qualitative methods (EA at Master’s level, JPB with PhD training) and experience in health systems research in Uganda. Transcripts were coded manually, and categories were grouped into themes and sub-themes through an iterative process.

#### Researcher positionality and reflexivity

The lead author (EA) is a Ugandan public health specialist who had previously worked with the Lira District Health Office, bringing both insider knowledge of the context and potential assumptions about the challenges facing health workers. To address potential bias: (1) reflexive memos were written throughout data collection and analysis to document and examine personal assumptions; (2) peer debriefing sessions with co-authors not based in Lira (RK, ER) provided external perspectives; (3) member checking was conducted by presenting preliminary findings to a group of 10 participating nurses and 5 community members to verify interpretation accuracy; (4) triangulation between quantitative survey data, KIIs, and FGDs helped balance different perspectives.

### Data management

All data were managed following a pre-established data management plan approved by the ethics committee. Quantitative data were double-entered into EpiData version 3.1 by two independent data entry clerks to verify accuracy; discrepancies were resolved by referring to original questionnaires. The cleaned dataset was exported to STATA 14 for analysis and stored on a password-protected university server with daily backups.

Qualitative audio files were transferred from recording devices to a secure university server immediately after each session and deleted from recorders. Transcripts were anonymized by removing all personal identifiers (names, specific locations, facility names) and assigning unique participant codes (e.g., KI01-HCIV, KI05-Hosp, FGD-EN-03 for Erute North participant 3, FGD-LM-07 for Lira Municipal participant 7). Anonymized transcripts were stored separately from the codebook linking participants to their characteristics. All digital files were encrypted, and access was restricted to the research team. Physical documents (consent forms, field notes) were stored in locked cabinets at Makerere University School of Public Health.

Data will be retained for ten years after study completion (until May 2030) as required by the Uganda National Council for Science and Technology, after which all data will be securely destroyed.

### Ethical considerations

Ethical approval was obtained from the Makerere University School of Public Health Research and Ethics Committee and the Uganda National Council for Science and Technology (Ref: SS 1234). Administrative clearance was granted by Lira District Health Office. Written informed consent was obtained from all participants prior to data collection. Participants were informed of their rights to withdraw from the study at any time without penalty or impact on their employment or services received. No incentives were offered for participation to avoid coercion. To ensure confidentiality, all personal identifiers were removed from the datasets, and participants were assigned unique codes. Data were stored on password-protected computers with access restricted to the research team. Physical consent forms were stored separately from the anonymized data in locked cabinets at Makerere University School of Public Health. All methods were performed in accordance with the relevant guidelines and regulations, including the Declaration of Helsinki.

## Results

### Socio-demographic characteristics of respondents

A total of 156 nurses and midwives participated (response rate ~ 100%). By cadre, 98 (62.8%) were nurses (enrolled and registered) and 58 (37.2%) were midwives (enrolled and registered). As shown in Table 1, the majority were female (83.3%, *n* = 130), aged 18–30 years (39.1%, *n* = 61), and married (73.7%, *n* = 115). Most (72.4%, *n* = 113) held certificates (enrolled nurse/midwife), and 54.5% (*n* = 85) had 1–10 years of work experience. A plurality (40.4%, *n* = 63) worked at Health Centre IIIs, and 78.9% (*n* = 123) were based in rural facilities.

**Table 1:** Socio-demographic and facility characteristics of respondents by cadre (*n* = 156)

Characteristic	Total *N* (%)	Nurses (*n* = 98) n (%)	Midwives (*n* = 58) n (%)
**Age group**			
18–30 years	61 (39.1)	38 (38.8)	23 (39.7)
31–40 years	49 (31.4)	31 (31.6)	18 (31.0)
41–50 years	39 (25.0)	25 (25.5)	14 (24.1)
51–60 years	7 (4.5)	4 (4.1)	3 (5.2)
**Sex**			
Male	26 (16.7)	22 (22.4)	4 (6.9)
Female	130 (83.3)	76 (77.6)	54 (93.1)
**Highest qualification**			
Bachelor’s Degree	3 (1.9)	2 (2.0)	1 (1.7)
Diploma	40 (25.6)	24 (24.5)	16 (27.6)
Certificate	113 (72.4)	72 (73.5)	41 (70.7)
**Years of experience**			
1–10 years	85 (54.5)	53 (54.1)	32 (55.2)
11–20 years	61 (39.1)	38 (38.8)	23 (39.7)
21–30 years	8 (5.1)	6 (6.1)	2 (3.4)
> 31 years	2 (1.3)	1 (1.0)	1 (1.7)
**Facility level**			
Hospital	19 (12.2)	12 (12.2)	7 (12.1)
Health Centre IV	43 (27.6)	27 (27.6)	16 (27.6)
Health Centre III	63 (40.4)	40 (40.8)	23 (39.7)
Health Centre II	31 (19.9)	19 (19.4)	12 (20.7)
**Facility location**			
Urban	33 (21.2)	22 (22.4)	11 (19.0)
Rural	123 (78.8)	76 (77.6)	47 (81.0)

### Principal Component Analysis (PCA)

PCA reduced the 45 independent variable items to six components (C1-C6) with eigenvalues > 1, accounting for 85.9% of the total variance (Table 2). The rotated component matrix (Table 3) revealed the following key latent variables:

#### C1

“Poor Clinical Practice” (e.g., not consulting treatment guidelines).

#### C2

“Adherence to Systems & External Influence” (e.g., following standards, political interference in promotion).

#### C3

“Organizational Commitment & Resource Lack” (e.g., recommending the organization, lack of supplies, low effort).

#### C4

“Participatory Environment & Working Conditions” (e.g., decision-making, poor work environment, data use).

#### C5

“Unmet Training & Remuneration Needs” (e.g., training not based on appraisal, low pay).

#### C6

“Skills & Politicized Recruitment” (e.g., having required skills, political recruitment influence).

### The six PCA components mapped onto the conceptual framework’s domains as follows

- **Individual factors domain**: C1 (Poor Clinical Practice), C5 (Unmet Training & Remuneration Needs), and elements of C6 (Skills) represent individual attributes and behaviors.
- **Organizational factors domain**: C2 (Adherence to Systems & External Influence), C3 (Organizational Commitment & Resource Lack), C4 (Participatory Environment & Working Conditions), and elements of C6 (Politicized Recruitment) represent organizational strategy and constraints.
- **Community factors domain**: While no single component captured community factors exclusively, community-level influences emerged through variables within C2 (external influence) and C4 (working conditions, which include community interactions), and were further explored through qualitative findings.

This mapping confirms that the empirical data align with our theoretical framework while revealing that organizational factors are more statistically dominant in explaining variance than individual or community factors alone.

**Table 2:** Eigenvalues and variance explained by principal components

Component	Eigenvalue	Difference	Proportion	Cumulative
1	21.97	15.56	0.488	0.488
2	6.41	1.72	0.142	0.631
3	4.69	2.04	0.104	0.735
4	2.65	0.80	0.059	0.794
5	1.85	0.73	0.041	0.835
6	1.12	-	0.025	0.859

The key components are summarized in Table 3; the full rotated component matrix is available in Supplementary file 3

**Table 3:** Rotated component matrix of factors affecting performance (key loadings ≥ |0.28|)

Component	Interpretive Label	Key Variables with High Loadings	Direction
C1	Poor Clinical Practice	Not consulting treatment guidelines	Negative
C2	Adherence to Systems & External Influence	Adhere to performance standards; Political interference in promotion	Mixed
C3	Organizational Commitment & Resource Lack	Recommend organization; Lack of supplies; Low effort	Mixed
C4	Participatory Environment & Working Conditions	Participate in decision-making; Unfavorable work environment	Positive/Negative
C5	Unmet Training & Remuneration Needs	Training not based on appraisal; Low pay	Negative
C6	Skills & Politicized Recruitment	Have required skills; Political influence in recruitment	Mixed

Note: Full rotated component matrix is available in Supplementary file 3

### Performance levels across dimensions

The average scores for performance dimensions were: Competency (3.03 ± 0.44), Productivity (3.01 ± 0.47), Availability (2.61 ± 0.68), and Responsiveness (2.97 ± 0.60). Categorization into percentile levels (Table 4) revealed that half (50.0%) of the respondents had competency levels in the 0–50% range. Productivity (60.3%), Availability (51.3%), and Responsiveness (75.0%) levels were predominantly in the 51–75% range.

**Table 4:** Performance level categorization by dimension

Performance Dimension	0–50%	51–75%	76–100%
Competency	50.0%	48.1%	1.9%
Productivity	26.9%	60.3%	12.8%
Availability	45.5%	51.3%	3.2%
Responsiveness	20.5%	75.0%	4.5%

### Predictors of performance: Linear regression analysis by dimension

Multiple linear regression models were run with each performance dimension and the overall performance score as dependent variables, and the six PCA components as independent variables. The consolidated results are presented in Supplementary file 4. Key findings are summarized below:

Overall performance (R² = 0.882): Components C1 (β= -0.011, *p* = 0.006), C2 (β = 0.064, *p* < 0.001), C3 (β = 0.020, *p* = 0.023), C4 (β = 0.120, *p* < 0.001), and C5 (β= -0.036, *p* = 0.001) were significant predictors.

Competency (R² = 0.835): Significantly predicted by C1 (β= -0.010, *p* = 0.012), C2 (β = 0.066, *p* < 0.001), C3 (β = 0.039, *p* < 0.001), and C4 (β = 0.080, *p* < 0.001).

Productivity (R² = 0.559): Significantly predicted by C1 (β= -0.029, *p* < 0.001), C2 (β = 0.030, *p* = 0.006), C3 (β = 0.044, *p* = 0.005), and C4 (β = 0.131, *p* < 0.001).

Availability (R² = 0.902): Significantly predicted by C2 (β = 0.090, *p* < 0.001), C4 (β = 0.131, *p* < 0.001), C5 (β= -0.060, *p* < 0.001), and C6 (β= -0.020, *p* = 0.004).

Responsiveness (R² = 0.951): Significantly predicted by C2 (β = 0.070, *p* < 0.001), C4 (β = 0.139, *p* < 0.001), and C5 (β= -0.047, *p* < 0.001).

### Integrated qualitative findings by performance dimension

Thematic content analysis of 20 key informant interviews and three focus group discussions yielded three overarching themes, each with sub-themes, which converged with and enriched the quantitative findings. Table 5 presents representative quotes for each theme, organized by participant type to demonstrate the range of perspectives captured. All quotations presented are verbatim from study participants collected during the research period.

While many challenges were reported across all facility types, important variations emerged by facility level, location, and ownership. Resource inadequacy was most acute in Health Centre IIs and IIIs, where 85% of respondents reported frequent stock-outs of essential medicines, compared to 40% in the regional referral hospital. Rural facilities (*n* = 123 respondents) faced additional challenges: 78% reported lack of staff accommodation, 65% lacked safe water supply, and 72% had unreliable electricity, compared to urban facilities where these figures were 24%, 18%, and 15% respectively.

Differences also emerged between government and PNFP facilities. PNFP facility staff (*n* = 28) reported better availability of medicines (65% reported consistent supply vs. 32% in government facilities) and more regular supervision (monthly vs. quarterly). However, PNFP staff reported lower salaries (average 30% less than government counterparts for equivalent qualifications) and less job security due to short-term contracts.

Political interference in recruitment was reported as a more significant problem in government facilities (78% of respondents) than in PNFP facilities (12%), reflecting the different governance structures.

**Table 5:** Representative quotes by theme and participant category

Theme	Sub-theme	Participant Category	Representative Quote
Organizational factors	Resource inadequacy	FGD participant, Erute South (FGD-ES-04)	*“The work environment is a big problem here*,* the workspace is inadequate with many cracks on the walls and floors*,* patients sleep on floors*,* lack of ventilation*,* excessive noise*,* inappropriate lighting*,* poor social services*,* lack of personal protective equipment*,* and this is really frustrating for us.”*
	Resource inadequacy	Key Informant, HC IV in-charge (KI08-HCIV)	*“Due to inadequate supply of medicines and supplies*,* lack of medical equipment and poor working conditions across all health facilities*,* it is difficult to assess the competencies of professional nurses and midwives. However*,* some of them seem to be competent.”*
	Poor leadership & work environment	FGD participant, Lira Municipal (FGD-LM-02)	*“Here*,* there is poor network*,* and when you want to communicate you have to search for a proper network that can be stable and also there are lots of conflicts among nurses due to poor communication*,* mostly during the handover of duty.”*
	Poor leadership & work environment	Key Informant, District Health Office (KI03-DHO)	*“The manager does not have enough authority to change these policies and the structure; therefore*,* if you cannot change the policies and the structure*,* you cannot execute a decision easily*,* and this then affects performance negatively.”*
Individual & political economy	Low morale and motivation	Key Informant, Senior Nursing Officer (KI15-Hosp)	*“Professional nurses and midwives are stressed and burnt out due to heavy workload*,* poor remuneration and low motivation that will affect their customer care attitude.”*
	Political interference	Key Informant, Hospital Matron (KI12-Hosp)	*“There is also political influence and interference. Some of these people who are recruited may be related to some politicians*,* and they want them to be working in certain areas*,* so it makes distribution of staff difficult.”*
Community-health worker dynamics	Poor health-seeking behaviors	Key Informant, HC III in-charge (KI05-HCIII)	*“I observed that because communities are very poor*,* sometimes people decide to come to the health facility when they are too late for health workers to save their lives. This reflects badly on the health workers because when patients die*,* they feel that they could have done something to save lives.”*
	Negative mutual perceptions	FGD participant, Erute North (FGD-EN-03)	*“The professional nurses and midwives treat us badly*,* like we are not human beings. They may pay no attention if someone dies compared with the traditional birth attendants who treat people humanely. In health centres/hospitals*,* they can slap you and say*,* ‘To avoid disturbances*,* let’s do the caesarean operation’. This brings fear and scepticism in using the health services.”*

Note: All quotations are presented verbatim from study participants. Minor grammatical corrections have been made only where necessary for readability while preserving original meaning; original audio recordings are available from the corresponding author for verification

## Discussion

This mixed-methods study provides a comprehensive analysis of multi-level factors affecting nurse and midwife performance in a Ugandan district. The findings demonstrate that performance is multi-dimensional, with distinct predictors for each dimension, and that individual, organizational, and community factors interact in complex ways to shape overall performance. The quantitative findings show that both individual attributes (e.g., clinical practice, commitment) and organizational factors (e.g., work environment, systems adherence) are strong, statistically significant predictors of all performance dimensions, explaining a large proportion of the variance (R² up to 0.951). This aligns with the strategic performance model by Noe et al. [25], which posits that individual characteristics and behaviors, shaped by the organizational context, are vital for performance.

### Competency: The gap between knowledge and practice

The low competency scores (50% in the bottom half) are particularly concerning and are linked to poor clinical practices like not consulting treatment guidelines (C1). This suggests a gap between knowledge and practice, possibly due to inadequate supervision, lack of access to guidelines, or high workload pressures. Similar findings have been reported in other low-resource settings, where clinical decision-making often deviates from standards due to systemic constraints [30, 31]. Our qualitative data illuminate this gap: “Some of the nurses, especially the newly posted ones, don’t consult the treatment guidelines. They prescribe based on what they remember from school, which may not be up to date” (KI08-HCIV), suggesting that competency is not simply a matter of individual knowledge but of workplace norms and accountability systems.

The strong association between competency and organizational factors, adherence to systems (C2), organizational commitment (C3), and participatory environment (C4), underscores that competency is shaped by the work context. This aligns with health system strengthening literature emphasizing that improving individual skills without addressing systemic constraints yields limited results [32, 33].

### Productivity: The impact of workload and resources

Productivity, measured by task completion and patient throughput, was significantly predicted by the same organizational factors (C2, C3, C4) as competency, with participatory environment (C4) showing the strongest effect (β = 0.131, *p* < 0.001). This reflects the reality that productivity depends not only on individual effort but on enabling conditions, available supplies, functional equipment, and supportive supervision [34, 35].

Qualitative findings revealed that productivity is severely constrained by understaffing: “The staffing level is about 60%, but the services are overwhelming. The available number of health workers is inadequate to provide quality, efficient, and effective health service deliveries” (KI12-HCIII). This staffing gap, based on 2002 norms that have not been updated to reflect population growth, represents a critical health systems failure requiring policy attention [36].

### Availability: Beyond physical presence

Availability, while encompassing physical presence at work, extends to having the necessary resources and support to provide care when present. The significant predictors C2 (adherence to systems), C4 (participatory environment), C5 (unmet training needs), and C6 (politicized recruitment) reveal that availability is fundamentally an organizational and political issue rather than merely an individual one.

The negative association with politicized recruitment (C6, β=-0.020, *p* = 0.004) is particularly striking. Our qualitative data explain this: “Some of these people who are recruited may be related to some politicians, and they want them to be working in certain areas, so it makes distribution of staff difficult” (KI03-DHO). This finding adds to evidence that decentralization in Uganda, while intended to improve local accountability, has sometimes facilitated political capture of human resource processes, undermining merit-based systems and professional autonomy [14, 37].

### Responsiveness: The patient-provider relationship

Responsiveness; treating patients with dignity, ensuring confidentiality, and being attentive to needs had the highest proportion in the 51–75% range (75.0%) but was significantly predicted by C2, C4, and C5. The negative association with unmet training needs (C5, β=-0.047, *p* < 0.001) suggests that when staff feel undertrained and undervalued, this manifests in how they treat patients.

Community perspectives provided critical insight into responsiveness failures: “The professional nurses and midwives treat us badly, like we are not human beings” (FGD-EN-03). This reciprocal breakdown in the patient-provider relationship, where communities’ negative perceptions reduce trust and utilization, and providers’ negative attitudes reflect low morale, creates a vicious cycle [38, 39]. Addressing responsiveness requires interventions targeting both health worker motivation and community engagement simultaneously.

### Engagement with Ugandan policy context

Our findings must be understood within Uganda’s existing policy framework. The Ministry of Health’s Human Resources for Health Policy (2019) explicitly aims to strengthen human resource management at the district level and ensure merit-based recruitment and deployment [40]. However, our findings reveal a persistent implementation gap between policy intention and district-level reality. The politicization of recruitment we document (C6) directly contradicts Sect. 4.2 of this policy, suggesting that political economy factors at the local level undermine national policy directives, a finding consistent with recent political economy analyses of Ugandan health systems [22].

Similarly, the Uganda National Minimum Health Care Package (UNMHCP) mandates specific staffing norms and service standards, yet our participants described severe understaffing (60% of required levels) and resource shortages that make UNMHCP implementation impossible. The Health Sector Development Plan 2015/16-2019/20 prioritized ‘improving health worker motivation and retention,’ yet our findings show low morale persists, indicating that current strategies (e.g., salary enhancements, housing allowances) may be insufficient without addressing underlying organizational and community-level factors.

The community engagement strategies outlined in the Health Sector Strategic Plan III, including Health Unit Management Committees and Village Health Teams, are designed to bridge community-facility gaps. However, our FGD participants’ descriptions of negative mutual perceptions and lack of community ownership suggest these mechanisms are functioning poorly in Lira District. This points to the need for not just policy presence but policy implementation support, including district-level capacity building, accountability mechanisms, and community empowerment initiatives, echoing findings of recent evaluations of community health worker programs in Uganda [41].

### Specific actionable recommendations by the stakeholder

#### For national policymakers (Ministry of Health)

- Revise the 2002 staffing norms to reflect the current population (estimated 30% increase since 2002), emerging diseases, and expanded service packages.
- Establish an independent health workforce commission to manage recruitment and deployment, removing these functions from district political structures. However, if decentralization is retained, mandate transparency, merit-based district panels with published criteria, independent oversight, and annual performance audits.
- Increase health sector financing to meet the Abuja Declaration target (15% of national budget) to address resource shortages.
- Mandate and fund regular supportive supervision with standardized tools and reporting requirements.

#### For district health managers

- Institute quarterly performance review meetings with facility in-charges using data from DHIS2.
- Establish transparent, merit-based promotion committees with clear criteria published annually.
- Implement a district-wide mentorship program pairing experienced with newer staff, particularly for clinical competencies.
- Ensure regular (monthly) facility inspections to address infrastructure gaps (water, power, accommodation).

#### For facility in-charges

- Conduct daily huddles to review staffing, supplies, and patient flow.
- Involve staff in developing duty rosters and facility work plans.
- Establish functional suggestion boxes and hold monthly staff meetings to discuss feedback.
- Strengthen Health Unit Management Committees through regular meetings and capacity building.

#### For community leaders and health unit management committees

- Conduct quarterly community dialogues to discuss health service experiences and expectations.
- Establish community recognition mechanisms for outstanding health workers.
- Mobilize community resources to address minor facility needs (e.g., cleaning, security) while advocating to the district for major investments.
- Monitor staff attendance and report persistent issues through established channels.

This study not only identifies key predictors of nurse and midwife performance but also carries important implications for future research. Theoretically, it reinforces the need to adapt existing performance models to account for political and community-level factors in decentralized health systems. Methodologically, it demonstrates the utility of mixed-methods approaches and PCA for distilling complex constructs in health workforce studies. Future studies should employ longitudinal designs to establish causal pathways and evaluate the impact of multi-level interventions on sustained performance improvement, as recommended by recent global health workforce research [42].

## Limitations of the study

Our study has several limitations. Its cross-sectional design limits causal inference. The performance measure was self-reported, which may be subject to social desirability bias. However, the use of a validated tool, high reliability scores, and triangulation with qualitative data strengthen validity. The focus on one district may affect generalizability, though the findings likely reflect challenges common to many rural, decentralized districts in Uganda and similar contexts.

### Temporal considerations

Data were collected between April 2017 and May 2018, and we acknowledge the time lag between data collection and publication. However, we argue the findings remain highly relevant for several reasons. First, a review of Lira District Health Office reports from 2019 to 2023 indicates that the challenges identified, such as staffing shortages, resource inadequacy, and political interference, persist and may have been exacerbated by the COVID-19 pandemic [43]. Second, the structural and systemic factors we identify (decentralization policies, staffing norms, resource allocation mechanisms) have not substantially changed since data collection. Third, similar findings continue to be reported in recent Ugandan health workforce studies [21, 22, 44], suggesting the issues are enduring. Nevertheless, readers should interpret findings with awareness of the data collection timeframe, and we recommend updated research to assess current conditions and the impact of post-pandemic health system changes.

### Instrument limitations

We acknowledge that some questions in the quantitative questionnaire (Supplementary file 1) may be double-barreled (e.g., combining “lack of equipment, medicine and supplies and few staffing” in a single item), which can make it difficult for respondents to answer accurately and for researchers to interpret which specific factor is being assessed. While PCA helps identify underlying latent variables, future research should use more precisely worded items that separate distinct constructs. Additionally, we acknowledge that some qualitative interview questions in the guides may have been phrased in ways that could be considered leading. While facilitators were trained to probe neutrally and follow participant responses rather than strictly adhere to question wording, this represents a limitation in the study design. Future qualitative research should employ more balanced questioning approaches and pilot test interview guides specifically for question neutrality.

## Conclusions

This study demonstrates that nurse and midwife performance in Lira District is shaped by a complex interplay of individual, organizational, and community factors that vary across performance dimensions. Three key messages emerge: First, organizational factors particularly participatory work environments, adherence to systems, and resource availability are the strongest and most consistent predictors of performance across all dimensions. Improving performance requires prioritizing investment in the work environment and management systems. Second, political interference in human resource processes (recruitment, deployment, promotion) fundamentally undermines meritocracy, staff morale, and accountability. This represents a critical governance failure that cannot be addressed through individual-level interventions alone. Third, the reciprocal breakdown in community-health worker relationships with negative attitudes on both sides creates a vicious cycle that reduces service utilization and provider motivation. Improving performance requires simultaneous investment in community engagement and trust-building.

**To address these challenges**,** we recommend**:

1. **Strengthen human resource governance**: If recruitment and deployment remain decentralized, establish transparent, merit-based district-level panels with clear, published criteria and independent oversight. Alternatively, if functions are centralized, create an independent health workforce commission to remove political interference. In either model, promotion committees must be performance-based and accountable to professional bodies rather than political actors.
2. **Invest in the work environment**: Ensure consistent availability of essential medicines, equipment, and functional infrastructure; track and address stock-out rates.
3. **Enhance supervision and support**: Reinvigorate supportive supervision focused on clinical competency; foster participatory facility-level leadership.
4. **Engage communities**: Implement structured community dialogue programs to improve health-seeking behaviors and rebuild trust.

Addressing these multi-faceted challenges requires a coordinated, system-strengthening approach from national policymakers, district managers, facility leaders, and communities to empower and enable nurses and midwives to perform to their full potential.

## Supplementary Information

Below is the link to the electronic supplementary material.

Supplementary Material 1

Supplementary Material 2

Supplementary Material 3

Supplementary Material 4

## Data Availability

The datasets used and/or analysed during the current study are available from the corresponding author on reasonable request.

## Abbreviations

DHIS2: District Health Information System
FGD: Focus Group Discussion
MOH: Ministry of Health
PCA: Principal Component Analysis
PNFP: Private Not-For-Profit
SDG: Sustainable Development Goals
UHC: Universal Health Coverage
UNMHCP: Uganda National Minimum Health Care Package
WHO: World Health Organization
