# Supplementary material for: Predictors of nurse and midwife performance in a Ugandan district: a mixed-methods study of individual, organizational, and community factors
Source: BMC Health Serv Res. 2026 Jun 8;26:1125. doi: 10.1186/s12913-026-14866-8 (PMC13474681; doi:10.1186/s12913-026-14866-8)
Supplement: Supplementary file 1 [file 12913_2026_14866_MOESM1_ESM.pdf]

QUESTIONNAIRES FOR PROFESSIONAL NURSES AND MIDWIVES

I am Acheka Edmonton, a student of Makerere University perusing a master of public health. I am currently conducting a study on the topic "factors affecting the performance of professional nurses and midwives in health facilities in Lira district 'in partial fulfillment of the requirements for the award of Master of Public Health''. This questionnaire is purposely to collect information related to the study. I kindly request you to participate willingly in the study as may be required. Any information provided will be kept confidential and will only be used for academic purpose.
Thank you for accepting to participate in this study.

Section A: Personal and Health facility information

1. What is your gender? 1. Male [ ] 2. Female [ ]

2. Age of respondents in completed …………………

3. Tell me your marital status 1. Married [ ] 2. Single [ ] 3. Divorced 4. Others specify………………...

4. What is your qualification? 1. BSC Nursing [ ] 2. BSC Midwifery [ ], 3. Registered Nurse/Midwife [ ]. 4, Enrolled Nurse/midwife [ ].

6. I have ……. of experience in the health profession

7. The type of health facility where I work.1. Hospital [ ], 2. Health Centre IV [ ], 3. Health Centre III [ ], 4. Health Centre II [ ].

8. I have been working in this health facility for …….

9. Location of Health facility 1. Urban [ ], 2. Rural [ ]

Section B 1: Individual factors (individual attributes).

10. Please indicate how you perceive your job-related individual attributes by circling only one answer to each of the statements below as follows: 1= Strongly disagree; 2= Disagree; 3= Undecided; 4= Agree; 5= Strongly agree

Statements	Strongly disagree	disagree	Undecided	agree	Strongly agree
1. I have the required skills and abilities to perform my tasks in accordance to my job description.					

2. I usually assess my clients in our facility using Nursing process					
3. I always strictly followed Nursing procedures when handling clients.					
4. I do follow performance standards developed by MOH for implementation of nursing procedures in our health facility.					
5. All my assigned tasks are always accomplished within a given period.					
6. I have been exposed to health education, customer care and counseling training one or more times in a year.					
7. When handing patients, I always consult the available Uganda treatment guidelines and Standard operating procedures (SOP).					
8. In our health facility I am fully utilizing 5 s strategy in organizing cleanliness of my work place.					
9. I feel so motivated by the fringe benefits I am receiving as a worker in this facility.					
10. I feel so motivated on how my performance is being recognized as a worker in this facility					
11. I am so much impressing with Human resources policies, relating to career perspectives in my health facility.					
12. I participate in decision making concerning patients‟ care and production of health facility work plan.					

Section B 2: Individual factors (individual behaviors).

11. To what extent do you agree with the statements below concerning your individual behavior? Please circle only one answer for each as follows:

1= Strongly disagree; 2= Disagree; 3= Undecided; 4= Agree; 5= Strongly agree

Statement	Strongly disagree	Disagree	Undecided	Agree	Strongly agree
**1.** Explaining everything to every client is time wasting because of client's volume.					
2. I would recommend others to seek employment at this organization.					
**3.** There are workdays where I just don't put much effort into my work.					
4. It is very difficult to balance family issues with workload in our health facility.					
**5.** I sometimes don't report on duty due to other commitments.					
6. I have initiated better ways of performing my core tasks.					
7. I have made suggestions to improve the overall performance of the organization (e.g., suggesting changes to administrative procedures)					
8. Lack of equipment, medicine and supplies and few staffing has made assessment of clients' needs not very possible.					
9. I am aware that my task is to serve my clients to the best of my ability.					
10. I always take very little time in performing my clinical work due to patient's load.					

Section C 1: Organization factors (organizational strategy).

12. Please indicate the extent to which you agree with the statements below regarding your Organizational strategies by circling only one answer for each as follows:

1= Strongly disagree; 2= Disagree; 3= Undecided; 4= Agree; 5= Strongly agree

Statement	Strongly disagree	Disagree	Undecided	Agree	Strongly agree
1. My working environment is considered unfavorable for good performance					
2. The facility has a clear mission					
3. The facility goals and objectives are very clear					
4. The facility has clear performance measurement procedures.					
5. Support of staff in form of counseling at workplace is available.					
6. There is a good balance between professional nurses and midwives and their supervisors.					
7. The facility has at least 50% MOH policy guidelines available.					
8. The policies of this organization are consistent with the cultural beliefs of the community.					
9. The health facility is fully implementing the Uganda National Minimum Health Care Package (UNMHCP) 100%.					
10. Recruitment of new professional nurses and midwives is politically influenced (only consider son or daughter of the soil).					
11. Promotion of health workers is based on merit.					

12. The local political leadership is supportive of professional nurses and midwives working in their locality.					
13. The facility has organized capacity building plan for the health workers.					

Section C 2: Organization factors (situational constrains).

13. Please indicate the extent to which you agree with the statements below concerning Situational Constraints in your health facility by circling only one answer for each as follows:

1= Strongly disagree; 2= Disagree; 3= Undecided; 4= Agree; 5= Strongly agree

Statement	Strongly disagree	Disagree	Undecided	Agree	Strongly agree
1. This organization has a system for collecting and tracking staff performance data.					
2. My performance is evaluated based on my job description.					
3. Constructive feedback on performance appraisal results is provided on a regular basis.					
4. The professional nurses and midwives' performance data is analyzed and reviewed according to the set performance standards, indicators, and targets.					
5. The professional nurses and midwives are given opportunity to make comments on the results of their performance.					
6. Rewards and sanctions are based on performance results					
7. The analysis of employee's training needs is based on the performance appraisal reports.					

8. There are procedures to collect suggestions for performance improvement from the professional nurses and midwives.					
9. Our remuneration is less attractive compared to other similar organizations.					
10. Professional nurses and midwives participate in identifying their career development needs.					

Section D: Performance Dimensions (dependent variable)

14. Please indicate the extent to which you agree with the statements below regarding performance dimension s of the services you provide at this facility by circling only one answer for each as follows:

1= Strongly disagree; 2= Disagree; 3= Undecided; 4= Agree; 5= Strongly agree

Statement	Strongly disagree	Disagree	Undecided	Agree	Strongly agree
**Responsiveness**					
1. Clients are always happy with the friendly services offered by professional nurses and midwives.					
2. Clients are satisfied with the quality of services we provide.					
3. Clients are satisfied with the timeliness of the services.					
4. The complaints from stakeholders towards professional nurses and midwives are rare.					
5. The suggestion box in this facility is fully functional and on weekly basis it is open and information sheared.					
6. The stakeholders are satisfied with the health workers" cooperation.					

7. I clearly know the catchment areas and population of people in my health facility					
8. Health workers are always willing to address the clinical & emotional demands of the patients.					
9. Professional Nurses and Midwives get technical support supervision, mentorship and coaching to improve their performance.					
10. My attitude towards the care of patients is good.					
**Availability**					
1. This organization has a retention policy and other policies with clear strategies.					
2. I am always available when my services are required.					
3. This facility has adequate numbers of health workers to deliver the services.					
4. The rural facilities are as well staffed as the urban ones.					
5. This facility has an attendance register which is well managed by every staff member.					
6. The feedback report from the attendance register is shared by HUMC, Sub- county authority and all the staff members.					
7. The report from the attendance register is being utilized properly to drive good performance.					
8. In this facility, there is enough and constant supply of medicines and supplies.					

9. The facility has enough functional medical equipment (vital signed equipment, and MCH equipment)					
10. This facility has enough staff accommodation.					
11. This facility has safe and constant water supply					
12. This facility has proper lighting system to facilitate night duty.					
13. This has all the social service nearby that can support heath worker's family.					
14. This facility has a functional HUMC in place.					
15. The workload in this facility is manageable					
16. In this facility the patient waiting time is short.					
17. The communities have full support for health service delivery in this facility.					
**Productivity**					
1. My productivity is measured according to the number of patients I attend to					
2. This organization has indicators for measuring staff productivity.					
3. There is well updated performance monitoring chart displayed in an open place.					
4. This facility has monthly performance review meetings					
5. Professional nurses and midwives in this facility are so much demotivated with the health system factors that influences their performance					

6. Professional nurses and midwives in this facility are so much demotivated with community factors that influence their performance.					
7. I spend most of my time at work attending to the patients.					
8. My skills are suited for the type of work I do.					
9. My productivity is increased by the availability of drugs & equipment.					
10. The management structures in this facility encourage the performance of health workers.					
**Competencies**					
1. I am confident about my ability to do my job.					
2. I always follow and consult my job description weekly.					
3. I always improve my knowledge and skills through continuous professional education.					
4. In my facility, it very difficult to use my knowledge & skills to improve safety of patients due some other factors					
5. In this facility, I do timely referrals of patients in case of management above my level.					
6. In this facility, maintaining good customer care is challenge.					
7. In this facility, I do organize duty monthly roaster and other time schedules for activities.					
8. I am so innovative in this facility, see the evidence.					

9. I am able to use the available communication technology to support patient care (computer and phone).					

Summary results for key informants' interviews

1 What are the most important aspects or factors that affect the performance of professional nurses and midwives in your district?

2 Professional nurses and midwives 'behaviors have an important contribution towards delivery of services in their respective health facilities. How do you describe the behavior of professional nurses and midwives in your district?

3 May you describe health workers in terms of: Availability, Competence, Responsiveness and Productivity?

4 What could be done to improve the performance of professional nurses and midwives in this Lira district?

INTERVIEW GUIDE FOR FGD FOR COMMUNITY PERCEPTION ON NURSE'S PERFORMANCE.

1 Tell me the community supports that are given to professional nurses and midwives to improve their performance of at your health facility?

2 In opinion, what do you think influences the health seeking behaviors of your community?

3 Kindly comment on the services you receive from professional nurses and midwives at your facility?

4 What are the common cultural practices and traditions that affect the performance of professional nurses and midwives at your health facility?

5 Kindly comment on the medicine and supplies you receive from your health facility?

6 Has there been effective and efficient service delivery provided by professional nurses and midwives at your facility?

7 What are the possible solutions to improve health service delivery at your health facility?
