# Supplementary material for: Predictors of nurse and midwife performance in a Ugandan district: a mixed-methods study of individual, organizational, and community factors
Source: BMC Health Serv Res. 2026 Jun 8;26:1125. doi: 10.1186/s12913-026-14866-8 (PMC13474681; doi:10.1186/s12913-026-14866-8)
Supplement: Supplementary file 2 [file 12913_2026_14866_MOESM2_ESM.docx]

**Supplementary file 2: Table S1.** Uganda health facility classification system and service packages

| **Facility Level** | **Administrative Unit** | **Catchment Population** | **Staffing Norm (Recommended Minimum)** | **Service Package** | **Typical Location** |
| --- | --- | --- | --- | --- | --- |
| **Health Centre II (HC II)** | Parish | 5,000 - 10,000 | • Enrolled Nurse (2) • Enrolled Midwife (1) • Nursing Assistant (2) • Health Assistant (1) | • Outpatient curative services (common illnesses) • Antenatal care (basic) • Immunization (outreach) • Health promotion and education • Community outreach and home visiting • Referral of complicated cases • No inpatient services • No surgical services | Rural (parish level) |
| **Health Centre III (HC III)** | Sub-county | 20,000 - 30,000 | • Senior Clinical Officer (1) • Clinical Officer (2) • Registered Midwife (2) • Enrolled Nurse (4) • Laboratory Technician (1) • Nursing Assistant (3) • Health Assistant (1) • Records Assistant (1) | • All HC II services • Inpatient care (general and maternity) • Maternity services (normal deliveries, basic emergency obstetric care) • Basic laboratory services (malaria, TB, HIV, urinalysis, stool analysis) • Expanded immunization services • Family planning services • Basic emergency care • Health management information systems | Rural (sub-county headquarters) |
| **Health Centre IV (HC IV)** | County | 100,000+ | • Senior Medical Officer (1) • Medical Officer (1) • Senior Clinical Officer (2) • Clinical Officer (3) • Senior Nursing Officer (1) • Registered Midwife (4) • Enrolled Nurse (6) • Laboratory Technologist (2) • Pharmacy Technician (1) • Radiographer (1) • Anaesthetic Officer (1) • Health Inspector (1) • Records Officer (1) • Support staff | • All HC III services • Emergency surgery (including caesarean sections) • Blood transfusion services • Comprehensive emergency obstetric care • Inpatient services (medical, surgical, pediatric) • Diagnostic services (X-ray, ultrasound) • Laboratory services (advanced) • Supervision of lower-level facilities (HC IIs and IIIs) • Outpatient specialist clinics • Health management and administration | Rural town or peri-urban |
| **General Hospital (District Hospital)** | District | 500,000+ | • Senior Medical Superintendent (1) • Medical Officers (4-8) • Senior Clinical Officers (4) • Clinical Officers (6-10) • Principal Nursing Officer (1) • Senior Nursing Officers (4) • Registered Nurses (15-20) • Registered Midwives (10-15) • Enrolled Nurses (20-30) • Laboratory Technologists (4-6) • Pharmacists (2) • Radiographers (2-3) • Anaesthetic Officers (2-3) • Dental Officers (1-2) • Physiotherapists (1-2) • Health Educators (1) • Records Officers (3-4) • Administrative staff • Support staff | • All HC IV services • Comprehensive inpatient and outpatient specialist services • General medicine, surgery, pediatrics, obstetrics/gynecology • Dental services • Mental health services • Comprehensive laboratory and imaging services • Blood bank • Pharmacy services • Teaching and training (internship) • Supervision of health centres in district • District health management support | District headquarters (often urban) |
| **Regional Referral Hospital** | Region | 2,000,000+ | • Hospital Director (1) • Deputy Hospital Director (1) • Senior Consultants (multiple specialities) • Medical Officers (specialists) • Principal Nursing Officers • Senior Nursing Officers • Registered Nurses and Midwives • Allied health professionals (all specialities) • Full administrative and support staff complement | • All general hospital services • Specialized clinical services (cardiology, neurology, oncology, etc.) • Advanced diagnostic services (CT scan, MRI, advanced pathology) • Specialist surgical services • Intensive care units • Teaching and research • Supervision of district hospitals in region • Tertiary referral services | Major city/regional headquarters |
| **National Referral Hospital** | National | National population (45+ million) | • Full specialist complement • Teaching and research staff • All cadres at maximum levels | • Comprehensive super specialist services • National reference laboratory • Advanced diagnostic and treatment services • Teaching and research (medical school) • Policy advisory role | Capital city (Kampala) |
