# Supplementary material for: Predictors of nurse and midwife performance in a Ugandan district: a mixed-methods study of individual, organizational, and community factors
Source: BMC Health Serv Res. 2026 Jun 8;26:1125. doi: 10.1186/s12913-026-14866-8 (PMC13474681; doi:10.1186/s12913-026-14866-8)
Supplement: Supplementary file 3 [file 12913_2026_14866_MOESM3_ESM.docx]

**Supplementary file 3: Table S2 Full rotated component matrix from PCA.**

**Table S2(a); Rotated Principal Components Analysis**

Principal components/correlation	Number of obs	=	156

Rotation: orthogonal varimax (Kaiser off) Rho	=	0.8594

**Component |	Variance	Difference	Proportion	Cumulative**

*Comp1* |	16.6329	9.79726	0.3696	0.3696

*Comp2* |	6.83567	1.42377	0.1519	0.5215

*Comp3* |	5.41191	.890251	0.1203	0.6418

*Comp4* |	4.52166	1.75645	0.1005	0.7423

*Comp5* |	2.7652	.259182	0.0614	0.8037

*Comp6* |	2.50602	0.0557	0.8594

To interpret each principal component, the magnitude and direction of the coefficients for the original variables were considered, in that the larger the absolute value of the coefficient, the more important the corresponding variable in calculating the component. The cutoff value for the absolute coefficient (Eigen vector) was set at 0.28, and any value less than 0.28 is indicated by blanks. Table S1, supplementary file 2: 1(b) shows.

**Table S2: Supplementary file 3 (b) Eigen vectors analysis of correlation matrix**

| **Components** | | | | | | |
| --- | --- | --- | --- | --- | --- | --- |
| **Variable Items** | **Comp 1** | **Comp 2** | **Comp 3** | **Comp 4** | **Comp 5** | **Comp 6** |
| **Individual factors** |  |  |  |  |  |  |
| Have required skills |  |  |  |  |  | 0.3094 |
| Adhere to performance set standard |  | 0.3906 |  |  |  |  |
| Not Consulting treatment guidelines when writing prescriptions | 0.2838 |  |  |  |  |  |
| Participate in decision making |  |  |  | 0.4343 |  |  |
| Recommend others to join organization |  |  | 0.3648 |  |  |  |
| Put less effort on their jobs some days |  |  | -0.3892 |  |  |  |
| Training needs not based on performance appraisal |  |  |  |  | 0.5283 |  |
| Suggest improvement ideas |  | 0.3464 |  |  |  |  |
| Remuneration is less attractive |  |  |  |  | 0.5298 |  |
| **Organizational factors** |  |  |  |  |  |  |
| Promotion not based on merit |  | 0.3209 |  |  |  |  |
| Politicians are not supportive |  | 0.3206 |  |  |  |  |
| unfavorable working environment |  |  |  | 0.4557 |  |  |
| Facilities implement UNMHCP. |  | 0.2920 |  |  |  |  |
| Lack of equipment, medicine and supplies and few staffing. |  |  | 0.3737 |  |  |  |
| Recruitment of nurses and midwives are politically influenced |  |  |  |  |  | 0.5763 |
| Performance data analyzed based on indicators |  |  |  | -0.3317 |  | 0.3914 |

*Rotated components (blanks are abs (loading) <0.28*
