# Supplementary material for: Predictors of nurse and midwife performance in a Ugandan district: a mixed-methods study of individual, organizational, and community factors
Source: BMC Health Serv Res. 2026 Jun 8;26:1125. doi: 10.1186/s12913-026-14866-8 (PMC13474681; doi:10.1186/s12913-026-14866-8)
Supplement: Supplementary file 4 [file 12913_2026_14866_MOESM4_ESM.docx]

**Supplementary file 4: Table S3 Consolidated linear regression results showing predictors of all performance dimensions.**

**Table S3:** Multiple linear regression model predicting performance dimensions (n=156)

| Performance dimensions | Predictor | Coefficient (β) | Std. Error | t-value | p-value | 95% CI Lower | 95% CI Upper | Significance |
| --- | --- | --- | --- | --- | --- | --- | --- | --- |
| **Overall performance** (R^2^=0.882) |  |  |  |  |  |  |  |  |
|  | Comp1 | -0.011 | 0.004 | -2.81 | 0.006 | -0.020 | -0.003 | *** |
|  | Comp2 | 0.064 | 0.006 | 10.56 | 0.000 | 0.052 | 0.076 | *** |
|  | Comp3 | 0.020 | 0.009 | 2.30 | 0.023 | 0.003 | 0.037 | ** |
|  | Comp4 | 0.120 | 0.008 | 14.71 | 0.000 | 0.104 | 0.136 | *** |
|  | Comp5 | -0.036 | 0.010 | -3.49 | 0.001 | -0.056 | -0.016 | *** |
|  | Comp6 | -0.011 | 0.006 | -1.89 | 0.060 | -0.022 | 0.000 | * |
|  | Constant | 0.178 | 0.050 | 3.54 | 0.001 | 0.079 | 0.277 | *** |
| **Competency** (R^2^=0.835) |  |  |  |  |  |  |  |  |
|  | Comp1 | -0.010 | 0.004 | -2.53 | 0.012 | -0.019 | -0.002 | ** |
|  | Comp2 | 0.066 | 0.006 | 10.81 | 0.000 | 0.054 | 0.078 | *** |
|  | Comp3 | 0.039 | 0.009 | 4.52 | 0.000 | 0.022 | 0.057 | *** |
|  | Comp4 | 0.080 | 0.008 | 9.75 | 0.000 | 0.064 | 0.096 | *** |
|  | Comp5 | -0.012 | 0.010 | -1.16 | 0.249 | -0.032 | 0.008 |  |
|  | Comp6 | -0.011 | 0.006 | -1.98 | 0.050 | -0.023 | 0.000 | * |
|  | Constant | 0.203 | 0.051 | 4.01 | 0.000 | 0.103 | 0.302 | *** |
| **Productivity** (R^2^=0.559) |  |  |  |  |  |  |  |  |
|  | Comp1 | -0.029 | 0.007 | -4.00 | 0.000 | -0.043 | -0.014 | *** |
|  | Comp2 | 0.030 | 0.011 | 2.80 | 0.006 | 0.009 | 0.051 | *** |
|  | Comp3 | 0.044 | 0.015 | 2.88 | 0.005 | 0.014 | 0.074 | *** |
|  | Comp4 | 0.131 | 0.014 | 9.14 | 0.000 | 0.103 | 0.159 | *** |
|  | Comp5 | -0.025 | 0.018 | -1.38 | 0.171 | -0.061 | 0.011 |  |
|  | Comp6 | -0.003 | 0.010 | -0.33 | 0.740 | -0.023 | 0.017 |  |
|  | Constant | 0.179 | 0.088 | 2.04 | 0.044 | 0.005 | 0.353 | ** |
| **Availability** (R^2^=0.902) |  |  |  |  |  |  |  |  |
|  | Comp1 | -0.001 | 0.005 | -0.27 | 0.787 | -0.011 | 0.008 |  |
|  | Comp2 | 0.090 | 0.007 | 12.37 | 0.000 | 0.076 | 0.105 | *** |
|  | Comp3 | -0.006 | 0.010 | -0.54 | 0.590 | -0.026 | 0.015 |  |
|  | Comp4 | 0.131 | 0.010 | 13.35 | 0.000 | 0.112 | 0.151 | *** |
|  | Comp5 | -0.060 | 0.012 | -4.86 | 0.000 | -0.085 | -0.036 | *** |
|  | Comp6 | -0.020 | 0.007 | -2.93 | 0.004 | -0.034 | -0.007 | *** |
|  | Constant | 0.159 | 0.060 | 2.64 | 0.009 | 0.040 | 0.279 | *** |
| **Responsiveness** (R^2^=0.951) |  |  |  |  |  |  |  |  |
|  | Comp1 | -0.006 | 0.003 | -1.83 | 0.069 | -0.012 | 0.000 | * |
|  | Comp2 | 0.070 | 0.005 | 15.46 | 0.000 | 0.061 | 0.079 | *** |
|  | Comp3 | 0.002 | 0.006 | 0.37 | 0.714 | -0.010 | 0.015 |  |
|  | Comp4 | 0.139 | 0.006 | 22.64 | 0.000 | 0.127 | 0.151 | *** |
|  | Comp5 | -0.047 | 0.008 | -6.06 | 0.000 | -0.062 | -0.032 | *** |
|  | Comp6 | -0.009 | 0.004 | -1.98 | 0.050 | -0.017 | 0.000 | * |
|  | Constant | 0.171 | 0.038 | 4.54 | 0.000 | 0.096 | 0.245 | *** |

**Model statistics summary:**

| Dimension | Mean DV | SD DV | R^2^ | F-test | p-value | AIC | BIC | N |
| --- | --- | --- | --- | --- | --- | --- | --- | --- |
| Overall | 0.581 | 0.103 | 0.882 | 185.073 | <0.001 | -586.928 | -565.579 | 156 |
| Competency | 0.605 | 0.087 | 0.835 | 125.330 | <0.001 | -585.183 | -563.834 | 156 |
| Productivity | 0.601 | 0.093 | 0.559 | 31.419 | <0.001 | -411.689 | -390.340 | 156 |
| Availability | 0.523 | 0.136 | 0.902 | 229.778 | <0.001 | -529.764 | -508.415 | 156 |
| Responsiveness | 0.595 | 0.119 | 0.951 | 477.970 | <0.001 | -676.790 | -655.441 | 156 |

****p<0.01, **p<0.05, *p<0.1**

**Note:** Components derived from PCA: C1 (Poor Clinical Practice), C2 (Adherence to Systems & External Influence), C3 (Organizational Commitment & Resource Lack), C4 (Participatory Environment & Working Conditions), C5 (Unmet Training & Remuneration Needs), C6 (Skills & Politicized Recruitment).
